# Supplementary material for: Identification of photoperiod-induced specific miRNAs in the adrenal glands of Sunite sheep (Ovis aries)
Source: Front Vet Sci. 2022 Jul 22;9:888207. doi: 10.3389/fvets.2022.888207 (PMC9354845; doi:10.3389/fvets.2022.888207)
Supplement: Supplementary file 1 [file Data_Sheet_1.ZIP › Supplementary file/miRDeep2 file.zip!/miRDeep2 file/Know_Structure/SP42A1/oar-mir-655.pdf]

miRBase precursor : oar-mir-655
Total read count : 2012
oar-miR-655-5p read count : 5
oar-miR-655-3p read count : 2007
remaining reads : 0

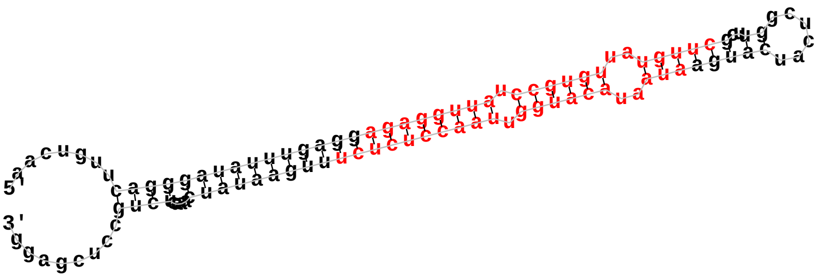

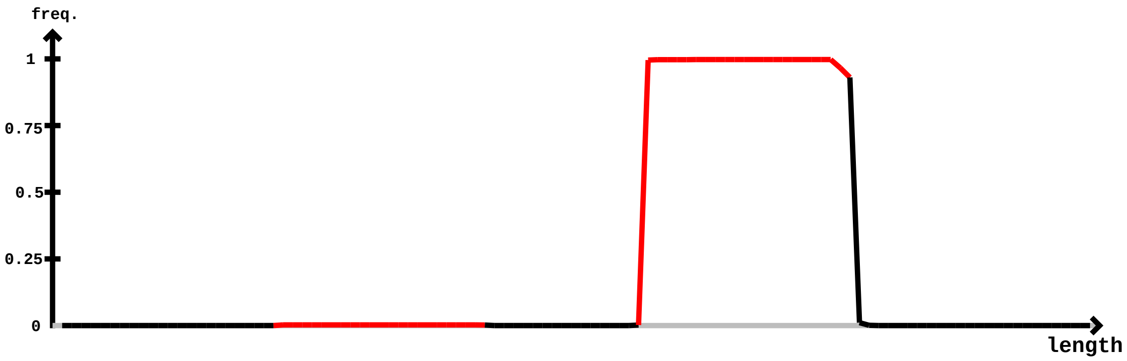


```
                                    oar-miR-655-5p
                                                                  oar-miR-655-3p
5'- aacuguucagggauauuugaggagagguuauccguguuauguucgcuuggcucaucaugaauaaauacaugguuaaccucucuuugaauaucaaacucugccucgagg   -3'   exp
    ...........(((((((((((((((((((.((((((..((((((..((.......))))))))..)))))).)))))))))))))))))))...(((......))).   reads   mm   sample
    ......................agagguuauccguguuaugu..................................................................   1       0    seq
    ......................agagguuauccguguuauguuc................................................................   4       0    seq
    ...........................................................aauaaauacaugguuaaccucu...........................   3       0    seq
    ...........................................................aauaaauacaugguuaaccucuc..........................   1       0    seq
    ............................................................auaaauacaugguuaaccucu...........................   60      0    seq
    ............................................................auaaauacaugguuaaccucuc..........................   71      0    seq
    ............................................................auaaauacaugguuaaccucucu.........................   1846    0    seq
    ............................................................auaaauacaugguuaaccucucuu........................   20      0    seq
    ............................................................auaaauacaugguuaaccucucuuu.......................   2       0    seq
    .............................................................uaaauacaugguuaaccucucu.........................   3       0    seq
    ..................................................................acaugguuaaccucucu.........................   1       0    seq
```
